# Biomedical Applications of Magnetically Functionalized Organic/Inorganic Hybrid Nanofibers

**DOI:** 10.3390/ijms160613661

**Published:** 2015-06-15

**Authors:** Hwa-Jeong Lee, Sang Joon Lee, Saji Uthaman, Reju George Thomas, Hoon Hyun, Yong Yeon Jeong, Chong-Su Cho, In-Kyu Park

**Affiliations:** 1 Department of Biomedical Sciences, BK21 PLUS Center for Creative Biomedical Scientists at Chonnam National University, Research Institute of Medical Sciences, Chonnam National University Medical School, Gwangju 501-757, Korea; E-Mails: hjl1510@gmail.com (H.-J.L.); kg2080@gmail.com (S.J.L.); sajiuthaman@gmail.com (S.U.); hhyun@jnu.ac.kr (H.H.); 2 Department of Radiology, Chonnam National University Hwasun Hospital, Chonnam National University Medical School, Gwangju 501-746, Korea; E-Mails: regeth@gmail.com (R.G.T.); yjeong@jnu.ac.kr (Y.Y.J.); 3 Department of Agricultural Biotechnology and Research Institute for Agriculture and Life Sciences, Seoul National University, Seoul 151-921, Korea

**Keywords:** nanofibers, magnetic nanoparticles, electrospinning, tissue engineering, Cancer therapy

## Abstract

Nanofibers are one-dimensional nanomaterial in fiber form with diameter less than 1 µm and an aspect ratio (length/diameter) larger than 100:1. Among the different types of nanoparticle-loaded nanofiber systems, nanofibers loaded with magnetic nanoparticles have gained much attention from biomedical scientists due to a synergistic effect obtained from the unique properties of both the nanofibers and magnetic nanoparticles. These magnetic nanoparticle-encapsulated or -embedded nanofiber systems can be used not only for imaging purposes but also for therapy. In this review, we focused on recent advances in nanofibers loaded with magnetic nanoparticles, their biomedical applications, and future trends in the application of these nanofibers.

## 1. Introduction

### 1.1. Nanofibers

Nanofibers are fibers with diameters less than 1000 nm [1]. Varying in length from tens of nanometers to a few microns, fiber features create surface topographies that affect various applications used in the nano- and biotechnology fields. Nanofibers tailored from natural and synthetic polymers have gained much interest because they are easy to synthesize and the structural, functional, and compositional properties of these nanofibers are tunable [2,3,4,5]. They can be produced by interfacial polymerization, electrospinning (ES), and electrostatic spinning. Carbon nanofibers are graphitized fibers synthesized under catalytic conditions. Among the possible techniques used to prepare nanofibers, such as phase separation, template synthesis, self-assembly, and drawing, ES is one of the most efficient, simple, and versatile methods owing to its relatively simple and cost-effective setup [6,7,8].

In the ES process, polymer nanofibers are produced by applying a strong electric field between a grounded target and the polymer solution. The polymer solution is fed with the use of a syringe pump through a metallic needle (spinneret) at a constant and controllable rate. The collector plate acts as the counter electrode on which the fibers are collected as a non-woven mesh or membrane. The process conditions and properties of the polymer solution influence the diameter of polymer nanofibers, which range from 10 to 1000 nm. The key advantage of producing polymer nanofibers with extremely small diameters is their large surface-to-mass ratio, high porosity, and superior mechanical performance [4,9]. Moreover, the functionality of the polymer nanofibers can be affected by the polymer molecules located at the surface of the polymer nanofibers, thus enabling customization of the nanofiber properties by tailoring the nanofiber surface compositions and morphologies. This ability to customize has led to diverse applications of nanofibers in wound healing, biosensors, drug delivery systems, medical implants, tissue engineering, dental materials [10,11,12], filtration membranes, military protective clothing, and other industrial applications [7,13].

Spinning polymer blends to create composite nanofibers allows for further tunability of nanofibers that can fulfill specific industrial requirements in terms of their material properties, thereby increasing their potential applications. Most recently, investigations have targeted the inclusion of other nanoscale structures within the nanofibers to produce structures with added functionality. For example, silver nanoparticles have been included in synthetic polymer nanofibers to produce a highly antimicrobial material [14]. In another study, emulsion droplets were successfully included in nanofibers. Self-assembled structures such as liposomes, micelles, and micro-emulsions have gained much attention in recent years [15,16,17]. They have been used as carrier systems for the delivery of antimicrobial agents, drugs, flavors, dyes, antioxidants, enzymes, and other functional compounds [18,19,20]. A combination of nanofibers and self-assembled structures, such as micelles, can thus create a novel delivery system with superior properties and many potential applications.

Owing to the advantageous features of nanofibers and magnetic nanoparticles (MNPs), many researchers have incorporated MNPs into biodegradable nanofibers to produce paramagnetic nanofiber scaffolds. To prepare these composite nanofibers, one of the most commonly used techniques is the mixing of dry inorganic powder with a polymeric solution followed by ES, although the nanocomposites formed are not stable and tended to agglomerate. To overcome this problem, various surface treatments have been used including salinization, polymer coating, and grafting. In addition to this type of surface coating, dispersion of Fe_3_O_4_ nanoparticles in the nanofibers has been performed in both water and organic solvents, as well as sodium citrate and oleic acid, although the complete dispersion of Fe_3_O_4_ nanoparticles was not achieved due to these incompatible interfaces [21].

### 1.2. Hybrid Nanofiber System

The addition of inorganic components to the polymer system facilitates the preparation of nanofibers with specific functionalities. A recent patent described an approach for preparing hybrid nanofibers by adding nanoparticles into the polymers [22]. In this patent, nanoparticle dispersion was easily carried out, and a porous structure was also created using salt dissolution. Antibacterial nanofibers utilizing Ag as an antibacterial agent have been prepared using ES [23]. To prepare the hybrid nanofibers by ES, the chemical precursor of silver NPs, AgNO_3_, was mixed with cellulose acetate (CA) or polyacrylonitrile (PAN) solution. The ES process was then followed by photo-reduction to form the silver NPs within the nanofibers formed. Chemical precursors of other metals were also used to obtain other types of hybrid nanofibers. For example, the chemical precursor of Pd was used to make poly(acrylonitrile-*co*-acrylic acid) (PAN-*co*-PAA)/Pd by ES [24]. In a separate study, gold NPs (AuNPs) were directly mixed with polymers prior to the ES process and subsequently electrospun (E-spun) to obtain hybrid nanofibers of poly(vinyl pyrrolidone) (PVP)/Au [25]. Other than with these metals, nanoparticles have also been prepared with functional metal oxides [26]. Table 1 summarizes the different inorganic components used for the preparation of hybrid nanofibers.

**Table 1:** Hybrid nanofibers and their applications.

Hybrid Nanofiber	Precursor and Polymer/Dopant	Potential Application	Reference
CO_3_O_4_	1. Cobalt acetate; 2. PVA/ H_2_O	Biomarker	[16]
Fe_3_O_4_	1. Iron (II) chloride; 2. Graft copolymer, poly(ethylene oxide) (PEO) or PVA	Drug carrier	[19]
CeO_2_	1. Cerium nitrate; 2. PVA/H_2_O	Catalyst	[27]
SiO_2_	1. Tetraethylorthosilicate; 2. HCl	Drug carrier	[28]
Ca_10_(PO_4_)_6_(OH, F)_2_	1. Ca(NO_3_)_2_; 2. P(C_2_H_5_O)_3_	Artificial bone	[29]
Ta_2_O_5_	1. Tantalum isopropyl oxide; 2. PVAC/DMF (or acetic acid)	Implant	[30]

**Figure 1:**
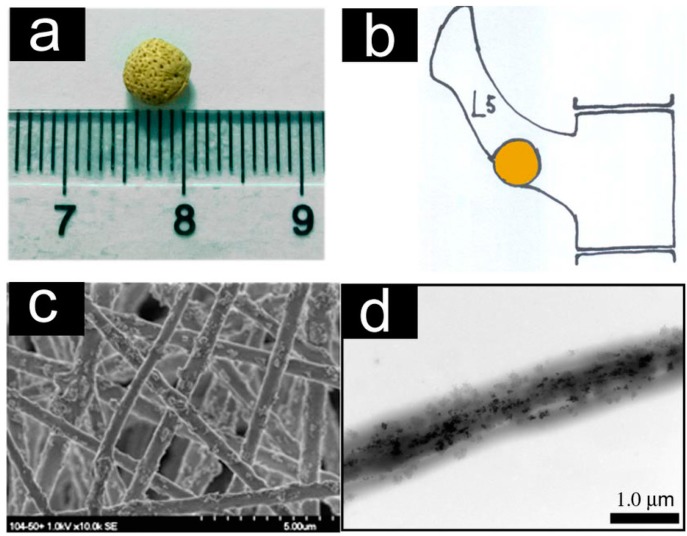
Characterization of the super-paramagnetic nanofibrous scaffolds. (**a**) The scaffold pellet with diameter of ~5 mm; (**b**): Schematic representation of the scaffold pellet implanted into the lumbar transverse defect of a white rabbit lumbar vertebral segment L5; (**c**) SEM image of the scaffold showing randomly tangled nanofibers with diameters ranging from 300 to 1000 nm; and (**d**) The TEM image of a single fiber. Reprinted with permission from [32].

Yang *et al.* reported a new method for preparing aligned fibrous arrays of composite magnetic nanofibers by ES [31]. As shown in Figure 1, nanofibrous arrays using polylactic acid (PLA) fibers can be applied in scaffolds without any structural changes. Also, the fiber morphologies remain intact after loading the MNPs. Moreover, their functionality can be controlled by adding selected types of NPs.

Recent studies have demonstrated the possibility of obtaining composite nanofibers by ES of ceramics and biopolymers. Hydroxyapatite (HA), a major component of bone, is a widely used bioceramic. Hybrid E-spun nanofibers containing HA as a bone regeneration implant material revealed high mechanical strength and good biocompatibility [28]. Observations from a scanning electron microscope (SEM) image revealed that the incorporation of HA did not change the required morphology and had a final structure consisting of smooth and interconnected nanofibers with high volume. A nanofibrous PLA/HA composite prepared by ES had good mechanical strength with fibers on the nanometer scale. This composite is promising as a temporary substrate for bone tissue regeneration. Inorganic compound-loaded nanofibers have been prepared using a combination of ES and the sol-gel process using common precursors such as SiO_2_, TiO_2_, and Al_2_O_3_ [33].

Polymer nanofibers loaded with Au, Ag, Pt, or Pd nanoparticles can be produced by ES with the addition of metal salt solutions as precursors. The diameters of the nanoparticles were in the range of 5 to 15 nm. These nanofibers have also been reported to have highly effective catalytic properties.

## 2. Methods to Prepare MNPs (Magnetic Nanoparticles)

MNPs are prepared via basic inorganic chemistry methods. Specifically, MNPs are prepared with magnetite, maghemite or iron alloys as the core magnetic material. MNPs can be prepared either by a single-step or a multi-step procedure, each of which has its advantages and disadvantages. There is no universal technique available for MNP synthesis. Commonly used methods for MNP synthesis will be briefly discussed in the following section.

### 2.1. Precipitation

One simple chemical method available for the preparation of MNPs is the precipitation method. It was developed to use aqueous solutions of iron (II or III) ions. Precipitation of MNPs can be accomplished using one of two methods: wet precipitation or co-precipitation. The wet precipitation method was developed first for MNPs preparation [34]. In the co-precipitation method, used for the preparation of iron oxide particles (Fe_3_O_4_), two stoichiometric solutions containing Fe^2+^ and Fe^3+^ ions are mixed with a base [35]. This co-precipitation method results in large nanoparticle sizes that are dependent on the pH of the solution. To synthesize MNPs successfully, the oxidation of the iron (II) precursor should be avoided because it leads to the conversion of Fe_3_O_4_ (magnetite) to Fe_2_O_3_ (maghemite), which might impair advantageous properties of Fe_3_O_4_ (magnetite) in its application as a contrast agent in magnetic resonance imaging (MRI). It has been shown that in the spinel structure of magnetite, cationic vacancies are in the octahedral positions, which result in a lower net spontaneous magnetization [36]. In particular, Basti *et al.* found that Fe_3_O_4_ magnetite provided stronger proton relaxivities in MRI than Fe_2_O_3_ maghemite [37]. As the process involves a large quantity of water, however, it is very difficult to scale-up the process [38]. One widely used method to effectively prevent oxidation is by bubbling N_2_, which leads to a reduction of the particle sizes. However, it is not easy to perform both precipitation and the addition of protective coating materials to the magnetic particles because maintaining the pH is laborious.

### 2.2. Reverse Micelle Formation

Micelle formation is a classic process in surfactant chemistry [39]. Normal micelles are usually synthesized in aqueous medium, whereas reverse micelles are formed in a mixture of a non-polar solvent and water. To produce iron oxide–based magnetic particles, the inorganic precursor of iron (III) chloride dissolved in aqueous medium is slowly added to the oily medium, followed by the addition of pH regulators [30,40,41,42,43]. The advantage of the reverse micelle method is to obtain organic-coated MNPs with controlled particle size. Also, it is possible to obtain inorganic-coated MNPs using reverse micelles [29,44,45,46,47,48]. The disadvantages of this method are that the remaining monomer hinders the coating of MNPs, it is difficult to scale-up the process due to the use of large amounts of organic solvent required, and it is not easy to prepare particles with a range of 20 to 500 nm because particle sizes depend on the size of the micelles [49,50,51,52,53,54].

### 2.3. Thermal Decomposition

Thermal decomposition is a popular method used in industry to synthesize MNPs because it does not use organic solvents [55]. The use of this method has led to advances in the preparation of metallic nanocrystals and semiconductors. Ferric and ferrous fatty acid complexes are widely used precursors of iron oxide super-paramagnetic particles because they are cheap, less toxic, and easier to scale-up for mass production [56]; However, the disadvantage of this method is that it is very difficult to control the particle size.

### 2.4. Liquid Phase Reduction

Strong reducing agents, such as NaBH_4_ and LiAlH_4_, are used to prepare MNPs through the reduction of magnetic or non-magnetic metal oxides to magnetic metal oxides. NaBH_4_ is the most commonly used reductant because it is soluble in both methanol and water [57,58,59,60]. The advantages of hybrid MNPs produced by this method are that they are very active, even under mild conditions, and can penetrate the polymer coating; however, they are difficult to handle due to their sensitivity to moisture.

## 3. Preparation of MNP-Functionalized Nanofibers

The development of MNP-functionalized nanofibers has attracted interest due to their potential use in scientific and industrial applications. The most widely used method for the preparation of MNP-loaded nanofibers is ES.

**Figure 2:**
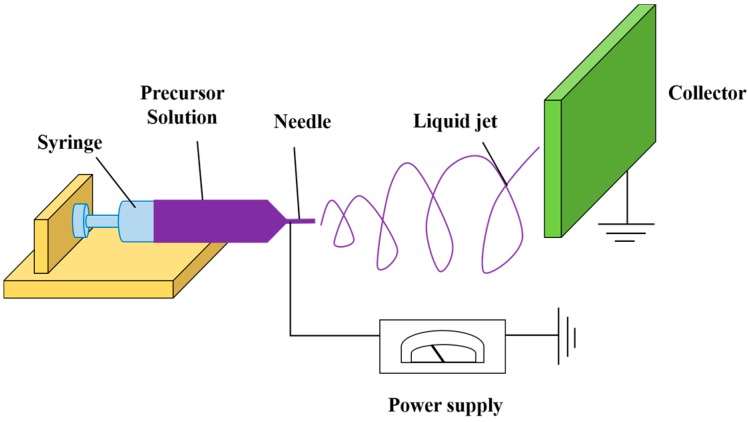
Schematic representation of the ES setup showing arrangement for syringe, precursor solution, needle, liquid jet, collector, and a power supply.

The first documented use of electro-hydrodynamics to modify the shape of a liquid meniscus under the influence of an electric field was reported by William Gilbert in the late 16th century [61]. In particular, he noticed that, when a suitably electrically charged piece of amber was brought near a droplet of water, it formed a cone shape and small water droplets were ejected from the tip of the cone. This was, in fact, the first recorded observation of electrospraying. The process of electrospinning (ES), developed by Anton Formhals in the thirties and forties of the last century [62,63,64] can be viewed as a special case of electrospraying. Larsen *et al*. [65] was the first to combine electrospinning with sol-gel methods to design nanofibers from inorganic oxides and hybrid materials. ES is a very simple method to produce nanofibers of a diameter range of 3 nm to 10 μm [66,67]. The technique mainly relies on the electrostatic repulsion of surface charges on the charged polymer solution and other variables, such as the solution flow rate, solution concentration, applied voltage magnitude, and the distance between the needle and the collector [68,69]. As shown in Figure 2, the ES setup involves a high-voltage direct current (DC) supply, a syringe pump, and a grounded collector. The basic ES technique involves the application of voltage on the polymeric droplet, thereby charging the droplet, followed by Taylor cone formation of a charged fiber jet, and accumulation on the grounded collector. Synthetic or natural polymers are widely used in making electrospun nanofibers [70], as well as the combination of synthetic and natural polymers, such as alginate/chitosan composite fibers [71]. Luong-Van *et al.* [72] prepared MNP-loaded poly(ε-caprolactone) (PCL) and MNP-functionalized poly(lactic-*co*-glycolide) (PLGA) nanofibers using ES. The size and morphology of magnetically-functionalized E-spun nanofibers can be controlled by changing the polymer concentration. Singh *et al.* reported the development of magnetic nanofibrous scaffolds composed of PCL and MNPs for bone regeneration [73]. In this study different weight ratio % of MNPs were distributed in PCL solutions up to 5 to 20 *w*_t_ % and subsequently E-spun into nonwoven nanofibrous webs. It was demonstrated that the fiber diameter was dependent on the MNPs added which in turn changes the electrical conductivity and viscosity of the solution. Other studies [74,75,76,77] have also used synthetic polymers like poly(l-lactic acid) (PLLA) and polyurethane to make electrospun nanofibers. Furthermore, Fan *et al.* [78] prepared PAN/Fe_2_O_3_ nano-composite fibers by suspending the Fe_2_O_3_ nanoparticles in PAN/DMF solution, followed by ES. The addition of MNPs to the precursor solution for the preparation of magnetic nanofibers can be beneficial because the spatial distribution of MNPs can be confined within the range of 200–500 nm in a one-dimensional structure (without stacking of the nanoparticles in the radial direction) [79]; however, size deformation of the nanofibers may occur due to the change of viscosity resulting from the addition of the MNPs to the precursor solution. Multi-functional polystyrene-based nanofibers with embedded MNPs have also been obtained in a single-step ES process [80].

## 4. Biomedical Applications of MNP-Functionalized Nanofibers

Nanofibers functionalized with MNPs have gained attention because of their potential applications (Table 2), including the use in sensors, tissue regeneration scaffolds, and drug delivery systems [81,82]. Many kinds of magnetic nanofibers have been synthesized, although they have not been applied in many practical applications. In the following section, several applications of nanofibers loaded with MNPs are discussed.

**Table 2:** Applications of nanofibers loaded with MNPs.

Source of Nanofiber	Kind of MNPs	Technology	Application	*In Vitro* and *in Vivo*	Reference
Chitosan / poly(vinyl alcohol) (PVA)	Fe_3_O_4_	ES	Bone regeneration	MG63 human osteoblast–like cells	[83]
Hydroxyapatite (HA) nanoparticles and poly(lactic acid) (PLA)	Super-paramagnetic Fe_2_O_3_ nanoparticles	ES	Bone tissue formation and remodeling in rabbit defects	White rabbit model of lumbar transverse defects	[32]
Poly(ε-caprolactone) (PCL)	MNPs	ES	Bone regeneration	Osteoblastic cells and subcutaneously implanted in rats	[73]
Hydroxyapatite (HA)	MNPs	Immersion of MNPs into HA scaffold	Bone repair	ROS 17/2.8 and MC3T3-E1 cells	[84]
Magnetic poly(l-lactic acid) (PLLA)	Fe_3_O_4_	ES	Enhanced effects on cell attachment and proliferation	MC3T3-E1	[85]
Poly(d,l-lactic acid) (PDLLA)	Superpara-magnetic iron oxide nanoparticles (SPIONs)	ES	Cell proliferation and induction of the cell orientation	Osteoblast cells	[86]
Chitosan	E-CHS-Fe_3_O_4_	ES	Hyperthermia treatment of tumor cells	HFL1 and caco-2 cells	[87]
Cross-linked chitosan	Fe_3_O_4_	ES by iminodiacetic acid (IDA)	Reduction of tumor cell proliferation	Tumor cells	[88]
Polystyrene (PS) and poly(styrene-*co*-maleic anhydride) (PSMA)	Magnetic NP-nanofibers (NF)	ES with surface-embedded T cell receptor ligand	Isolation and activation of primary CD3^+^ T lymphocytes	Lymph nodes harvested from C57BL/6 mice	[89]
Porous hydroxyapatite composite	Up-conversion of luminescent and MNPs	ES	Indomethacin, T1 magnetic resonance imaging (MRI) contrast agents, and luminescent nanoparticles	MC 3T3-E1 cells	[90]
Hydroxyapatite nanocrystals within PCL	Doped with gadolinium (Gd)	ES	*In situ* monitoring of bone tissue regeneration by MR	Human mesenchymal stem cells	[91]
Amphiphilic peptide	Macrocyclic Gd (III)	β-sheet amino acid sequence	MRI	*Tibialis anterior* muscle of a murine model	[92]

### 4.1. Scaffold for Bone Regeneration

Bone regeneration by tissue engineering is very important for bone defects resulting from tumor resection, trauma, and skeletal abnormalities. Even though many kinds of scaffolds used for tissue engineering for bone repair have been investigated [93,94], the development of a successful scaffolding material is needed to satisfy clinical requirements.

Using the ES technique, Meng *et al.* prepared a novel nanofibrous scaffold composed of maghemite super-paramagnetic MNPs, hydroxyapatite, and PLA [32]. The nanofibrous pellet was implanted into the lumbar transverse defect of a white rabbit. The rabbits were raised in rabbit cages fixed with permanent magnets to provide a static magnetic field after surgery. The observed enhancement of tissue regeneration in the lumbar defect when the magnetic field was applied pointed to a novel strategy to improve bone tissue regeneration based on MNPs-functionalized nanofibers.

**Figure 3:**
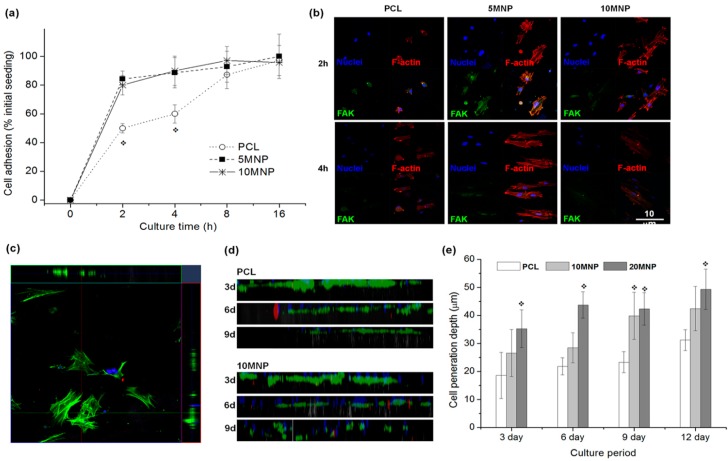
MC3T3-E1 cell adhesion and penetration tests on the nanofiber scaffolds. (**a**) Initial cell adhesion level on the nanofiber scaffolds during culture for up to 16 h, presented as % initial seeding. Significantly higher levels of cell adhesion noticed on the PCL-MNP scaffolds *vs.* PCL (* *p* < 0.05); (**b**) Cell adhesion morphology taken from confocal microscopy of immunofluorescent stained cells at 2 h and 4 h of culture; nuclei in blue, F-actin in red and FAK in green; (**c**–**e**) Cell penetration assay through the nanofiber scaffolds; exemplar image showing that z-stacking of immunofluorescence-stained cells (F-actin in green and nuclei in blue) were unfolded on xz- and yz-planes to reveal 2D constructed images (**c**), which were then combined to complete construction of depth profile of cells on 2D plane view (**d**, compared images of PCL and 10MNP samples at a culture period of 3, 6 or 9 days), and the quantification of depth profile (**e**, shown average positions of cell penetration depth), showing significant improvement in cell penetration within the nanofibers incorporating MNPs. Reprinted with permission from [73].

The resulting nanofiber webs became more hydrophilic with improved mechanical properties due to the addition of MNPs into the PCL scaffold. The addition of MNPs resulted in magnetic properties of the nanofibrous scaffolds, typical for weak ferromagnetic or super-paramagnetic materials, as well as increased hydrophilicity, accelerated scaffold degradation, and apatite-forming ability. When osteoblast cells were cultured on the MNP-loaded nanofibers versus on pure PCL nanofibers, initial cell adhesion and penetration increased. Furthermore, rats implanted with MNP-loaded PCL nanofibers showed significantly better bone regeneration with minimal adverse reactions. Singh *et al.* [73] incorporated MNPs in PCL and studied adhesion, spreading, and penetration of mesenchymal stem cells (MSCs) (Figure 3), which revealed enhanced cell penetration depth with higher content of MNPs.

Another study [85] reported MNP-functionalized PLLA using trifluoroethanol (TFE) as a cosolvent. The composite nanofibers formed by ES showed paramagnetic properties with minimum cytotoxicity and enhanced cell attachment.

Hydroxyapatite (HA), used extensively for bone regeneration, has also been applied as a scaffold. In one study [84], MNPs were loaded into the pores of HA to make a magnetic biomimetic scaffold for bone repair. This magnetic HA scaffold had good cell adhesion, differentiation, and proliferation ability.

### 4.2. Cancer Therapy

Hyperthermia-based cancer therapy using MNPs has recently gained wide interest; However, the application of free MNPs has several limitations due to low solubility, poor cancer targeting, and leakage of MNPs from the tumor location. Therefore, current strategy involves MNP-loaded electrospun nanofibers for localized hyperthermia-based tumor treatment.

In a recent study, 50-nm iron oxide nanoparticles (IONPs) were loaded into polystyrene (PS) electrospun nanofibers to allow for repeated heating by applying an alternating magnetic field (AMF) with minimum IONP leakage. IONP-loaded PS nanofibers were made by spontaneous ES of IONPs after dispersing of IONPs in a PS solution containing a mixture of tetrahydrofuran and dimethylformamide (1:3 volume ratio) to form uniform nanoscale electrospun fibers. Most of the human SKOV-3 ovarian cancer cells attached to IONP-loaded PS nanofiber mats through applying an AMF were dead as a result of the cancer hyperthermia effect [88].

In another study [89], electrospun MNP-loaded chitosan nanofibers were prepared by two different methods: a) direct adsorption of MNPs into nanofibers by immersion of MNPs into a chitosan solution, and b) direct immersion of chitosan in an Fe^2+^/Fe^3+^ solution and co-precipitation of MNPs by ammonium hydroxide. However, there were not many differences in the morphologies and *in vitro* hyperthermic effects in Caco-2 cells between the nanofibers made by the two different methods; although, cross-linking of the MNP-loaded chitosan nanofibers with gluteraldehyde during ES was performed. Similarly, in another study [90], cross-linked chitosan nanofibers using iminodiacetic acid (IDA) were prepared using the co-precipitation method to increase the loading of the MNPs into the nanofibers. In addition, Ganesh *et al.* loaded MNPs into thermoplastic poly(ethylene terephthalate) (PET) nanofibers [91]. MNP-loaded nanofibers have potential to be used as cancer hyperthermia therapy through the application of an AMF.

### 4.3. Tissue Engineering

Tissue engineering is the substitution of any human organ or tissue with artificial functional materials using a combination of biology, medicine, and engineering. Recently, Preslar *et al.* manipulated target cells in a scaffold using physical means, such as magnetic force [92]. Nanofibers loaded with MNPs used as the scaffold play an important role in wound healing [93], because the MNPs in the nanofiber scaffold assemble the tissue and aid tissue formation by magnetic force. Using this technique, the magnetic force-based tissue engineering and mechano-transduction methods can control cellular signaling, artificial blood vessel development, and bone tissue formation [85,94]. In addition, this method is an effective way to mimic signal transduction *in vivo* and convert extracellular mechanical stress to intracellular chemical cues [94]. Furthermore, MNP-loaded nanofiber scaffolds have been applied in cell sheet engineering for skin tissue formation through the creation of multi-layered keratinocytes by magnetic force [95].

## 5. Conclusions and Future Prospects

In this review, magnetically functionalized E-spun nanofibers have been discussed from the standpoint of biomedical applications, including scaffolds, regenerative medicine, and cancer therapy. These fibers combine inherent characteristics of standard E-spun nanofibers, such as high porosity, high surface-area-to-volume ratio, flexibility, and ease of fiber production, with unique and advantageous magnetic properties.

In the future, magnetic E-spun nanofibers will likely be used as a functionalized scaffold in tissue engineering because MNP-loaded nanofiber scaffolds have *in vitro* osteogenesis and tissue compatibility and the ability to regenerate bone *in vivo* due to their increased hydrophilicity, accelerated degradation, ability to form apatites, and enhanced mechanical properties by magnetic force, suggesting that they can be used as a new class of bone regenerative materials. In addition, electrospun nanofiber mats with a conformal coating on the surface of the mats may be used as an ideal wound-dressing material because they are antibacterial, nontoxic, non-antigenic, permeable for gaseous exchange, resistant to shearing forces (due to their elasticity), and are highly water-absorbent. However, the use of highly engineered electrospun nanofiber mats tailored for wound healing in the clinic requires further study. Furthermore, nanofiber systems may be used in the future as an alternative approach to gastro-retentive drug delivery systems for enhanced bioavailability and the controlled release of drugs because they provide prolonged contact time with the gastric mucosa, controlled release of the drug, and good stability.
